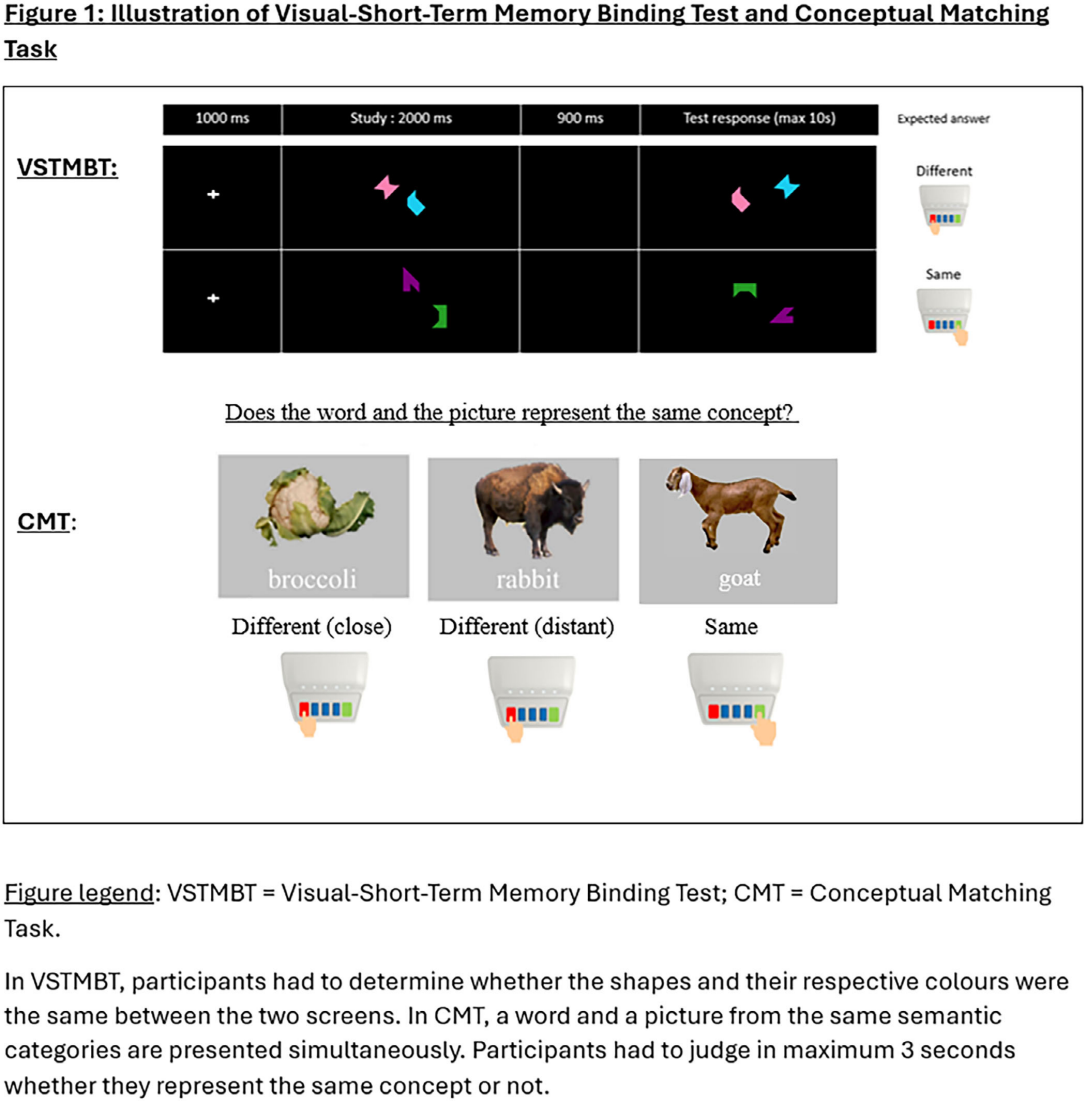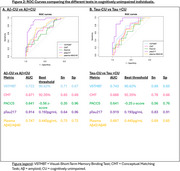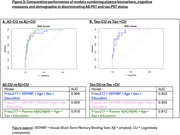# Discrimination tasks increase the value of plasma *p*‐tau217 to predict presymptomatic Alzheimer's disease

**DOI:** 10.1002/alz70862_109967

**Published:** 2025-12-23

**Authors:** Lara Huyghe, Lisa Quenon, Jean‐Louis Bayart, Lise Colmant, Emilien Boyer, Thomas Gérard, Yasmine Salman, Gabriel Besson, Laurence Dricot, Renaud Lhommel, Adrian Ivanoiu, Christine Bastin, Emma Delhaye, Bernard J Hanseeuw

**Affiliations:** ^1^ Institute of Neuroscience, UCLouvain, Brussels Belgium; ^2^ Department of Neurology, Saint‐Luc University Hospital, Brussels Belgium; ^3^ Institute of Neuroscience, UCLouvain, Brussels, 1200 Belgium; ^4^ Departement of Laboratory Medicine Cliniques Saint Pierre, Ottignies Belgium; ^5^ GIGA‐CRC, University of Liège, Liège Belgium; ^6^ Saint‐Luc University Hospital, Brussels Belgium; ^7^ Massachusetts General Hospital, Harvard Medical School, Boston, MA USA

## Abstract

**Background:**

AD can be diagnosed using amyloid (Aβ) and tau‐PET imaging. These techniques are expensive and invasive, making them hardly practical for screening in clinically unimpaired (CU) populations. Research is therefore focused on developing more affordable methods to detect pre‐symptomatic AD pathology. Specific cognitive metrics and diverse blood‐based biomarkers, including soluble phosphorylated tau (*p*‐tau) species, have emerged as promisingly reflecting an underlying aggregated Aβ or tau pathology in the brain. However, head‐to‐head comparisons between these measures are relatively scarce and their additive value to detect incipient AD pathology is largely undetermined. We aimed to investigate the value of plasma *p*‐tau217 and cognitive tasks assessing perceptual or conceptual discrimination to identify CU individuals with PET‐imaging evidence of AD pathology.

**Method:**

Eighty CU older adults underwent a blood test for plasma *p*‐tau217, Aβ40 and Aβ42, [^18^F]‐MK6240 tau‐PET, [^18^F]‐Flutemetamol or [^11^C]‐PIB amyloid‐PET, 3D‐T1 brain MRI, a neuropsychological assessment including PACC5, the Conceptual Matching Task (CMT), and the Visual‐Short Term Memory Binding Test (VSTMBT). These tasks evaluate conceptual and perceptual discrimination abilities respectively (Figure 1). Participants were classified based on Aβ status [Centiloid<20: Aβ‐ (*n* = 55) and Centiloid>20: Aβ+ (*n* = 25)] or tau‐PET visual Braak stage [Braak=0: Tau‐CU (*n* = 65) and Braak>0: Tau+CU (*n* = 15)]. ROC curves were computed to determine optimal threshold, sensitivity, and specificity of each test. We also performed logistic regression models with receiver operating characteristic (ROC) curves to determine the accuracy of plasma biomarkers and cognitive measures in differentiating Aβ‐PET and tau‐PET status.

**Result:**

*p*‐tau217 was the measure with the larger AUC for detecting incipient amyloidosis or tauopathy as evidenced using PET (AUC=0.91). Among cognitive measures, VSTMBT was the most sensitive and accurate measure for detecting Aβ (AUC=.722) and tau‐PET positivity (AUC=0.743) (Figure 2). The combined use of plasma and cognitive markers (Figure 3) allows predicting AD pathology in clinically unimpaired individuals (AUC=0.93 for Aβ and AUC =0.95 for tau). Specifically, the addition of very subtle cognitive deficits to pTau217 allowed increasing sensitivity for positive tau‐PET from 0.83 to 1.0 (with specificity=0.91).

**Conclusion:**

The combined use of plasma markers and cognitive measures looks promising for the early diagnosis of Alzheimer's disease.